# *APOE4* Promotes Tonic-Clonic Seizures, an Effect Modified by Familial Alzheimer’s Disease Mutations

**DOI:** 10.3389/fcell.2021.656521

**Published:** 2021-03-16

**Authors:** Lorissa Lamoureux, Felecia M. Marottoli, Kuei Y. Tseng, Leon M. Tai

**Affiliations:** ^1^Biological Resources Laboratory, University of Illinois at Chicago, Chicago, IL, United States; ^2^Department of Anatomy and Cell Biology, College of Medicine, University of Illinois at Chicago, Chicago, IL, United States

**Keywords:** Alzheimer’s disease, apolipoprotein E, seizure, amyloid beta, sex

## Abstract

Seizures are emerging as a common symptom in Alzheimer’s disease (AD) patients, often attributed to high levels of amyloid β (Aβ). However, the extent that AD disease risk factors modulate seizure activity in aging and AD-relevant contexts is unclear. *APOE4* is the greatest genetic risk factor for AD and has been linked to seizures independent of AD and Aβ. The goal of the present study was to evaluate the role of *APOE* genotype in modulating seizures in the absence and presence of high Aβ levels *in vivo*. To achieve this goal, we utilized EFAD mice, which express human *APOE3* or *APOE4* in the absence (EFAD−) or presence (EFAD+) of familial AD mutations that result in Aβ overproduction. When quantified during cage change day, we found that unlike *APOE3*, *APOE4* is associated with tonic-clonic seizures. Interestingly, there were lower tonic-clonic seizures in E4FAD+ mice compared to E4FAD− mice. Restraint handing and auditory stimuli failed to recapitulate the tonic-clonic phenotype in EFAD mice that express *APOE4*. However, after chemical-induction with pentylenetetrazole, there was a higher incidence of tonic-clonic seizures with *APOE4* compared to *APOE3*. Interestingly, the distribution of seizures to the tonic-clonic phenotype was higher with FAD mutations. These data support that *APOE4* is associated with higher tonic-clonic seizures *in vivo*, and that FAD mutations impact tonic-clonic seizures in a paradigm dependent manner.

## Introduction

Alzheimer disease (AD) is the most common form of dementia and is defined by cognitive decline, extracellular plaques containing amyloid-β (Aβ) and intraneuronal tangles of tau. In addition, accumulating evidence suggest that seizures are a common and important component of the AD phenotype (reviewed in Palop and Mucke, 2009; Friedman et al., 2012; Pandis and Scarmeas, 2012; Lam and Noebels, 2020). For example, the risk of clinical unprovoked seizures of unknown etiology is 6–10-fold higher in AD and between 1 and 60% of AD patients experience unprovoked seizures (Hauser et al., 1986; Risse et al., 1990; Romanelli et al., 1990; McAreavey et al., 1992; Mendez et al., 1994; Volicer et al., 1995; Hesdorffer et al., 1996; Amatniek et al., 2006; Lozsadi and Larner, 2006; Rao et al., 2009; Scarmeas et al., 2009; Bernardi et al., 2010; Irizarry et al., 2012; Imfeld et al., 2013; Vossel et al., 2013; Cheng et al., 2015; Sarkis et al., 2016). However, the incidence of seizures in AD is likely to be underestimated because AD patients also experience non-convulsive seizures that are often unrecognized due to their symptomatic overlap with other behavioral changes such as memory loss, hallucinations, anxiety, and confusion. In support of this concept, subclinical epileptiform activity has been observed in up to 40% of AD patients when assessed by electroencephalogram recordings (Vossel et al., 2013, 2016; Brunetti et al., 2020; Lam et al., 2020). Recent data also suggest that seizures occur in early, preclinical stages of dementia and accelerate disease progression (Cretin et al., 2016; DiFrancesco et al., 2017; Costa et al., 2019; Keret et al., 2020). Therefore, evaluating the role of AD risk factors in modulating seizure activity is a crucial step to establish whether there is link with disease progression.

*APOE* genotype is the greatest genetic risk factor for sporadic AD, with *APOE4* increasing risk up to 12-fold compared to *APOE3* (reviewed in Mahley et al., 2007; Liu et al., 2013; Flowers and Rebeck, 2020). The role of *APOE* in AD is extremely complex and includes modulation of functions both independent and dependent of AD pathology (particularly Aβ). For example, *APOE4* is associated with learning and memory dysfunction during aging, independent of AD (reviewed in Tai et al., 2016), which is recapitulated in *APOE* targeted replacement mice (Grootendorst et al., 2005; Villasana et al., 2006; Bour et al., 2008; Rodriguez et al., 2013; Tai et al., 2016; Thomas et al., 2017; Zaldua et al., 2020). Critically, there is an association between *APOE* genotype and seizures (Hirsch, 2007). With *APOE4* there is higher epilepsy risk (Liang et al., 2019), particularly post trauma (Diaz-Arrastia et al., 2003; Harden, 2004), an earlier age of onset for intractable seizures (Briellmann et al., 2000; Gambardella et al., 2005; Kauffman et al., 2010) and greater memory dysfunction in patients with chronic temporal lobe epilepsy (Gouras et al., 1997; Gambardella et al., 2005; Busch et al., 2007). Unfortunately, *in vivo* research on this topic is limited, with only one report of higher seizures with *APOE4* in *APOE*-targeted replacement mice (Hunter et al., 2012). This study highlights the ability of *APOE* to modulate brain function independent of AD pathology, but there is a strong link between *APOE4* and Aβ. In humans and mice that overproduce Aβ, with *APOE4* levels of all different types of Aβ (soluble, soluble oligomeric, intraneuronal, extracellular) are higher compared to *APOE3*. Aβ itself is also linked to higher seizures as evidenced by the higher incidence of seizures found in familial AD (FAD) patients (reviewed in Palop and Mucke, 2009). FAD accounts for 5% of all cases and is caused by mutations in proteins (the amyloid precursor protein, or presenilins) that result in higher Aβ production; 40–80% of FAD patients experience seizures (Palop and Mucke, 2009). Mouse models of FAD mutations also develop epileptic spiking consistent with partial seizures (Palop et al., 2007; Minkeviciene et al., 2009; Siwek et al., 2015; Gureviciene et al., 2019), have a lower seizure threshold to pentylenetetrazole (Del Vecchio et al., 2004) and have higher audiogenic seizures (Westmark et al., 2010; Kazim et al., 2017). However, it is currently unknown whether *APOE* genotypes modulate seizures in the presence of human Aβ (i.e., *APOE*/FAD mice), and if the combination of *APOE4* and Aβ results in higher seizures compared to *APOE4* alone. Addressing these questions could provide novel insight on the contribution of *APOE* genotype to seizures in AD-relevant contexts, and therefore provide the framework for future research focused on identifying the underlying mechanisms.

The goal of the present study was to evaluate the role of *APOE* genotype in modulating seizure incidence in the absence and presence of high Aβ levels *in vivo*. To achieve this goal, we utilized EFAD mice that express *APOE3* or *APOE4* in the absence (EFAD−) or presence (EFAD+) of Aβ overproduction. We recorded total seizure incidence during cage changes, evaluated whether handling restraint or auditory cues precipitated seizures and assessed seizure threshold to pentylenetetrazole.

## Methods

### Animals

All experiments procedures were approved by the Institutional Animal Care and Use Committee at the University of Illinois at Chicago. We used EFAD mice (Youmans et al., 2012), which were produced by crossing mice that express 5 Familial Alzheimer’s disease mutations (5xFAD; APP K670N/M671L + I716V + V717I and PS1 M146L + L286V, C57BL6/B6xSJL) with *APOE*-targeted replacement mice (*APOE*-TR, C57BL6). EFAD non-carrier mice are *APOE*^+/+^ 5xFAD^–/–^ (E4FAD−) and carriers are *APOE*^+/+^/5xFAD^±^ (EFAD+). All EFAD mice were at least 8 months old at the start of the study. Mice were group-housed in a 12–12-h light-dark cycle (lights off at 11 a.m. and on at 11 p.m.).

### Colony Observations of Seizures

Tonic-clonic seizures are relatively easy to identify in the mouse colony as the noise during an episode is unique and loud and were recorded in EFAD mice during routine once weekly cage change by trained members of the animal husbandry staff. Cages and the corresponding mouse (tail) were marked that displayed seizure behavior during cage change at the end of the light cycle. Tonic-clonic seizures were reported if they occurred when the cage was removed from the housing rack, during cage changing in the changing station, or once the cage was placed back on the housing rack.

### Acute Handling Restraint Stress-Induced Seizures

The prevalence of acute handling restraint stress-induced seizure behavior (tonic-clonic) was assessed in the same mice as those evaluated for seizures during cage change day. Mice were manually restrained for a maximum of 30 s using the scruffing technique that involves grasping the loose skin located around the dorsal aspect of the mouse’s neck. If a seizure began while in restraint, the mouse was placed into an empty cage to assess the duration of tonic-clonic seizure and recovery. If a seizure did not begin while in restraint, the mouse was placed in a new cage and observed for 1 min for seizure occurrence, prior to returning to the home cage.

### Audiogenic-Induced Seizures

A subset of EFAD mice were subjected to an audiogenic seizure protocol adapted from Yagi et al. (2005) to investigate seizure susceptibility triggered by sound. Briefly, mice were allowed to acclimate in the sound-attenuating cabinet (Ugo Basile) for 1 min prior to the presentation of four 11 kHz tone at an intensity of 105 dB for 20 s, with a 2 s interval. All behavioral changes were recorded using a video camera affixed to the chamber and evaluated off-line.

### Pentylenetetrazole (PTZ)-Induced Seizures

A single dose (60 mg/kg, s.c.) of pentylenetetrazole (PTZ, Sigma-Aldrich^TM^, St. Louis, United States; purity ≥ 99%) was used to evaluate seizure thresholds in the same cohort of mice exposed to the audiogenic protocol. After injection, mice were placed in acrylic boxes, and behavioral changes induced by PTZ were recorded for 30 min using a video camera above the testing arena and analyzed off-line by investigators blinded to *APOE* genotype and sex. This dose of PTZ typically induces a range of seizure-like behaviors from freezing and myoclonic twitches to tonic-clonic seizures (Brault et al., 2011; Garcia-Cabrero et al., 2013; Bezzina et al., 2015; Van Erum et al., 2020). Tonic-clonic seizures begin with freezing behavior alternating with myoclonic twitching of the forelimbs that progress to violent jumping and running. Mice with tonic-clonic seizures lasting greater than 3 min were euthanized. We also assigned a score to the types of seizures; 3 = tonic-clonic, 2 = freezing and 1 = no seizure.

### Statistics

**FIGURE 1:**
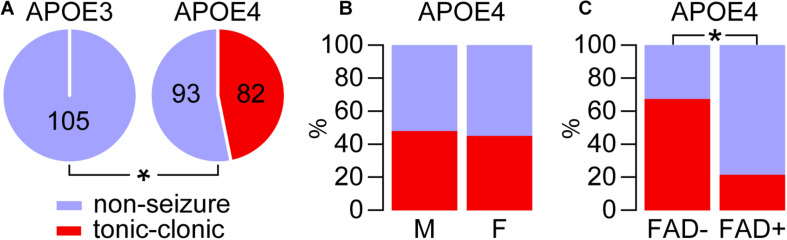
*APOE4* but not *APOE3* is associated with tonic-clonic seizures during cage change day. The incidence of tonic-clonic seizures was evaluated during cage change day over a 5-week period in mice that express *APOE3* or *APOE4* in the absence (E3FAD– and E4FAD–) and presence (E3FAD+ and E4FAD+) of five familial AD mutations. **(A)** Tonic-clonic seizures manifested in mice that express *APOE4*, but not *APOE3* (*p < 0.00001, E4FAD– and E4FAD+ vs. E3FAD– and E3FAD+ mice, Fisher Exact test). **(B)** There were similar seizure frequencies in male and female *APOE4* mice (*p* = 0.70, male E4FAD– and E4FAD+ vs. female E4FAD– and E4FAD+, Chi-square test). **(C)** Interestingly, there were higher seizures in the absence of FAD mutations in *APOE4* mice (*p < 0.0001, E4FAD– vs. E4FAD+ mice, Chi-square test). *n* = 23 male E3FAD–, 25 female E3FAD–, 58 male E4FAD–, 34 female E4FAD–, 28 male E3FAD+, 29 female E3FAD+, 46 male E4FAD+, 37 female E4FAD+ mice.

**FIGURE 2:**
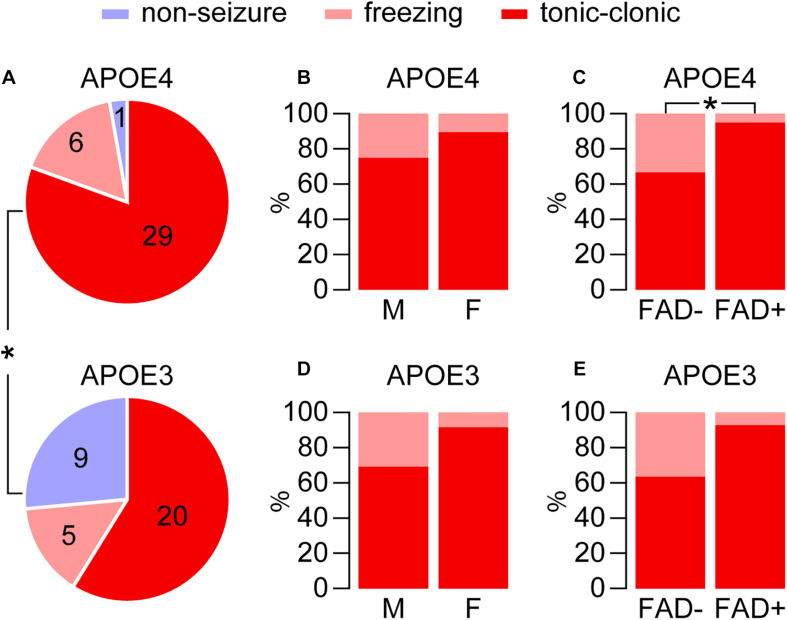
Higher seizures with *APOE4* for pentylenetetrazole-induced seizures. Seizure frequency was evaluated in EFAD mice after treatment with pentylenetetrazole (PTZ, 60 mg/kg), which resulted in a range of seizure-like behaviors from freezing and myoclonic twitches to tonic-clonic seizures. **(A)** Seizure behaviors were modulated by *APOE* genotype and were higher with *APOE4* in EFAD mice (*p < 0.028, E4FAD– and E4FAD+ vs. E3FAD– and E3FAD+ mice, Chi-square test). **(B)** With *APOE4* expression, in male (12/16) and female (17/19) mice there was a similar distribution of seizures (tonic-clonic compared to total, *p* = 0.26, male E4FAD– and E4FAD + vs. female E4FAD– and E4FAD+, Chi-square test). **(C)** However, there was higher distribution of tonic-clonic seizures in E4FAD+ (19/20) compared to E4FAD– (10/15) mice (*p = 0.028, E4FAD– vs. E4FAD+ mice, Chi-square test). With *APOE3* expression **(D)** tonic-clonic seizure frequency was also similar for each sex (*p* = 0.16, 9/13 male E3FAD– and E3FAD+ vs. 11/12 female E3FAD– and E3FAD+, Chi-square test). **(E)** Although not statistically significant, there was trend of higher tonic-clonic seizure incidence with FAD mutations (*p* = 0.07, 7/11 E3FAD– vs. 13/14 E3FAD+ mice, Chi-square test). *n* = 6 male E3FAD–, 11 female E3FAD–, 8 male E4FAD–, 8 female E4FAD–, 9 male E3FAD+, 8 female E3FAD+, 9 male E4FAD+, 11 female E4FAD+.

**FIGURE 3:**
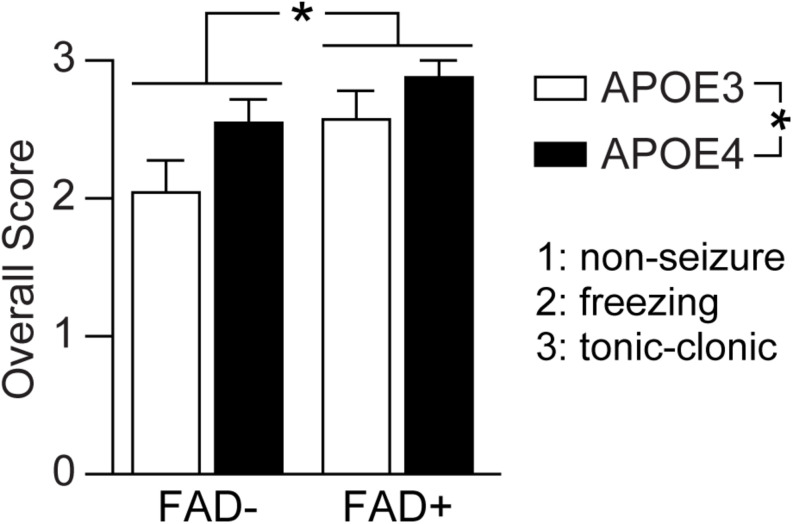
*APOE* genotype and FAD mutations independently modulate pentylenetetrazole-induced seizures. Seizure score was evaluated in EFAD mice after pentylenetetrazole (PTZ, 60 mg/kg) treatment using a scale of 3 (tonic-clonic), 2 (freezing) and 1 (non-seizure). When assessed by two-way ANOVA, *APOE* and FAD modulated seizure score, however there was no interaction. Data is expressed as mean ± S.E.M. Data evaluated by two-Way ANOVA. *F*(1, 65) = *APOE* genotype: 5.59, *p = 0.021; FAD: 6.32 *p = 0.014; *APOE* × FAD interaction: 0.331, *p* = 0.5670. *n* = 6 male E3FAD–, 11 female E3FAD–, 8 male E4FAD–, 8 female E4FAD–, 9 male E3FAD+, 8 female E3FAD+, 9 male E4FAD+, 11 female E4FAD.

In Figures 1 and 2 comparisons were made using Chi-squared test (GraphPad Prism version 8) to assess the incidence of seizure behaviors. Fisher’s exact test was utilized when one variable equaled zero. *p* < 0.05 was considered significant. We employed sequential statistical analysis, by first focusing on our primary research question of whether *APOE* genotype modulates seizure behaviors (*APOE3* vs. *APOE4*). We then evaluated the impact of sex (male vs. female), and FAD mutations (EFAD + vs. EFAD-) on seizures within each *APOE* genotype. In Figure 3 seizure score was evaluated via two-way ANOVA.

## Results

To identify how *APOE* genotype modulates behavior and brain function in aging and AD-relevant contexts, we utilize EFAD mice, which express human *APOE3* or *APOE4* in the absence (EFAD-) or presence (EFAD+) of FAD mutations that result in Aβ overproduction. Over the last few years, we started to observe seizures in mice expressing *APOE4*, especially after spending more time in the mouse colony during the dark/active cycles. Thus, the goal of this study was to systematically determine the effect of *APOE* on seizure frequency and thresholds in EFAD− and EFAD+ mice. Specifically, we utilized E3FAD−, E3FAD+, E4FAD−, and E4FAD+ mice, both male and female that were at least 8 months of age at the start of the study. The age cut-off was selected as anecdotally we had had not observed seizures in younger mice.

### *APOE4* but Not *APOE3* Is Associated With Tonic-Clonic Seizures During Cage Change Day

The types of seizures we observe in E4FAD− and E4FAD+ mice are tonic-clonic, which typically begins with freezing behavior, progresses to severe myoclonic twitching of the forelimbs with violent movements (uncontrolled jumping, running), followed by recumbency. Tonic-clonic seizures are readily identifiable, and after discussions with the animal husbandry staff, it became apparent that some EFAD mice had been seizing in response to the stimuli produced during routine cage change. Therefore, we sequentially evaluated the effect of *APOE* genotype, sex and FAD/Aβ on seizure frequency during weekly cage change over a 5-week period. All available mice of at least 8 months of age in the colony were utilized for this study: 23 male E3FAD−, 25 female E3FAD−, 58 male E4FAD−, 34 female E4FAD−, 28 male E3FAD+, 29 female E3FAD+, 46 male E4FAD+, 37 female E4FAD+ mice.

We first assessed whether *APOE* genotype was associated with higher seizure frequency, though combining data for FAD- and FAD+ mice for each genotype (Figure 1A). There were tonic-clonic seizures in 46.9% of mice that express *APOE4* (82 out of 175) compared to 0% that express *APOE3* (0 out of 105). We next evaluated whether within the *APOE4* genotype group (E4FAD− and E4FAD+), biological sex affected seizure frequency. We found that both male and female *APOE4* mice showed similar frequency of seizure occurrence (Figure 1B): 48.1% in males (50/104) vs. 45.1% in females (32/71). Thus, unlike some other readouts found in this model (Aβ levels, neuroinflammation, cerebrovascular function) (reviewed in Tai et al., 2017; Balu et al., 2019), there is no effect of sex on seizures with *APOE4* during cage changing. We next asked whether *APOE4* alone or *APOE4* and FAD mutations/Aβ levels contribute to tonic-clonic seizures (Figure 1C). Our data demonstrate that E4FAD− mice (67.4%, 62/92) are more likely to experience seizure behaviors than E4FAD+ mice (24.1%, 20/93 E4FAD+). This effect was found within each sex, as both male (60.3%, 35/58 E4FAD− vs. 32.6%, 15/46 E4FAD+, *p* < 0.01, Chi-square) and female (79.4% 27/34 E4FAD− vs. 13.5%, 5/37 E4FAD+, *p* < 0.0001, Chi-square) E4FAD− mice showed higher incidence of seizures when compared to E4FAD+ mice. Collectively, these data demonstrate that *APOE4* is associated with tonic-clonic seizures compared to *APOE3*, and that FAD mutations/Aβ levels are associated with a lower number of seizures in *APOE4* mice.

### Acute Handling Restraint and Auditory Stimulus Do Not Trigger Seizures in EFAD Mice

We next explored whether the effect of *APOE* genotype on tonic-clonic seizures can be elicited by acute stress alone. To this end, we conducted a standardized handling restraint test in all the mice that were evaluated for seizures during cage change day. During restraint handling, only 1.1% of mice that express *APOE4* (2 out of 175; 1 male E4FAD− and 1 female E4FAD−) showed seizures, whereas none of the *APOE3* mice (0/105) did. These data indicate that acute stress associated with handling is insufficient to induce seizures to the extent that we observed during cage change day with *APOE4* in EFAD mice.

An additional stressor that can induce seizures in mice, inducing FAD mice, is auditory stimuli, which we next evaluated in a subset of EFAD mice; 6 male E3FAD−, 11 female E3FAD−, 8 male E4FAD−, 8 female E4FAD−, 9 male E3FAD+, 8 female E3FAD+, 9 male E4FAD+, 11 female E4FAD+. We found that auditory stimulus alone is insufficient to induce seizures in any of the groups of EFAD mice tested. Together, these results suggest that acute stress due to handling and auditory stimuli do not mimic the number of seizures we observed with cage change day in EFAD mice.

### Higher Seizures With *APOE4* for Pentylenetetrazole (PTZ)-Induced Seizures

As neither E3FAD− or E3FAD+ had seizures during cage change day, our next goal was to characterize the effect of *APOE* genotype on seizure susceptibility after chemical induction using PTZ (60 mg/kg). For these experiments we used the same cohort of mice subjected to auditory stimulus testing.

We paralleled our analysis of cage change day seizures, through first evaluating the role of *APOE* genotype in modulating seizure frequency after PTZ injection (i.e., E4FAD− and E4FAD+ vs. E3FAD− and E3FAD+). As found in other studies (Brault et al., 2011; Garcia-Cabrero et al., 2013; Bezzina et al., 2015; Van Erum et al., 2020) PTZ administration resulted in a range of seizure-like behaviors from freezing and myoclonic twitches to tonic-clonic seizures. Overall, the incidence of the combined seizure behaviors was higher with *APOE4* compared to *APOE3* (97.2%, 35/36 E4FAD− and E4FAD+ mice vs. 73.5%, 25/34 E3FAD− and E3FAD+ mice, Figure 2A). In addition to exhibiting a higher frequency of tonic-clonic seizures (80.5%, 29/36 *APOE4* vs. 58.8%, 20/34 *APOE3*, *p* < 0.05, Chi-square test), only 1 out of the remaining 7 *APOE4* mice showed no seizures compared to 9 out of the 14 *APOE3* mice.

We next evaluated if sex or FAD genotype within the *APOE4* group (E4FAD− and E4FAD+) modulated PTZ-induced seizures. There was similar frequency of total seizure occurrence in both male (94.1%, 16/17) and female (100%, 19/19) *APOE4* mice (*p* = 0.47, male E4FAD− and E4FAD+ vs. female E4FAD− and E4FAD+, Fisher’s exact test). In addition, sex did not alter the distribution of seizures (tonic-clonic seizures compared to total seizures, 12/16 male, 17/19 female, Figure 2B). E4FAD− (93.8%, 15/16) and E4FAD+ (100%, 20/20) mice also exhibited comparable levels of total seizure occurrences (*p* = 0.44, E4FAD− vs. E4FAD+ mice, Fisher’s exact test). However, when evaluated as a distribution of total seizures, a higher proportion of E4FAD+ mice (95%, 19/20) developed tonic-clonic seizures when compared to the E4FAD− group (66.6%, 10/15) (Figure 2C).

As with *APOE4*, a similar frequency of total seizures was observed between male (86.7%, 13/15 male) and female *APOE3* mice (63.2%, 12/19 female) (*p* = 0.12, male E3FAD- and E3FAD+ vs. female E3FAD− and E3FAD+, Chi-square test) with similar seizure distribution (9/13 male, 11/12 female, Figure 2D). E3FAD− (64.7%, 11/17) and E3FAD+ (82.3% 14/17) mice also exhibited comparable levels of total seizures (*p* = 0.24, E3FAD− vs. E3FAD+ mice, Chi-square test). Although there was a higher frequency of tonic-clonic seizures in E3FAD + mice (92.8%, 13/14) compared to the E3FAD− group (63.6%, 7/11), this was not statistically significant (*p* = 0.07, Figure 2E). Our study may have lacked power to detect differences in PTZ-induced tonic-clonic seizures between E3FAD- and E3FAD + mice. Therefore, we performed additional analysis by assigning a score (3 = tonic-clonic, 2 = freezing and 1 = no seizure) to each mouse after PTZ injection (Figure 3). When assessed by two-way ANOVA, a significant main effect of *APOE* and FAD were detected, indicating that both variables modulated the behavioral effects of PTZ. However, there was no *APOE* x FAD interaction, supporting our initial analysis that after PTZ treatment, the incidence of seizures is higher with *APOE4* and with FAD mutations.

Collectively, these results demonstrate that compared to *APOE3*, *APOE4* is associated with higher incidence of PTZ-induced seizures. Further, in contrast to cage change day, our data indicate that FAD mutations/Aβ levels are associated with a higher distribution of tonic-clonic seizures in *APOE4*, and potentially *APOE3* mice.

## Discussion

Compared to *APOE3*, *APOE4* is associated with tonic-clonic seizures when assessed during cage change day and greater seizure incidence after PTZ injection. Although sex did not modulate seizure incidence in EFAD mice, there was an important impact of FAD genotype. For tonic-clonic seizures in *APOE4* mice, FAD mutations were associated with lower incidence when measured during cage change day, but a higher distribution after PTZ injections. Collectively, our data support that *APOE4*-associated seizures are an important component of the behavioral phenotype in aging-and AD-relevant mice. Thus, research focused on evaluating the cellular basis of these seizures could provide mechanistic insight onto how *APOE* and FAD mutations modulate neural circuit function and connectivity.

### Higher Seizure Incidence With *APOE4*: AD Relevance

Seizures have emerged as an important component of the AD phenotype, with multiple groups reporting higher seizure incidence in patients with dementia and AD (reviewed in Palop and Mucke, 2009; Friedman et al., 2012; Pandis and Scarmeas, 2012; Lam and Noebels, 2020). Rather than a cause of AD, seizures represent a manifestation of altered neuronal function, which may exacerbate brain dysfunction and disease progression caused by the complex repertoire of AD pathologies. Although tonic-clonic seizures could be managed with anti-epileptic medications, sub-clinical seizures may be overlooked in AD patients and the underlying causes of the neuronal hyperexcitability will remain. Thus, understanding the extent AD risk factors modulate seizures could enable mechanistic research on their underlying causes that in turn can be translated to effective biomarker and therapeutic applications for AD patients in the clinic. In a disease as complex as AD, the threshold for neuronal dysfunction to produce seizures may be modified by genetic and lifestyle risk factors. In this study, we report that seizures are higher with one of the greatest genetic risk factors for AD, *APOE4*, in both the absence and presence of FAD mutations (see section “Seizure Incidence Is Modified by FAD Mutations but Not Sex” for discussion on sex and FAD). These data are consistent with reports suggesting that epilepsy risk is higher with *APOE4* (Liang et al., 2019), including after trauma (Diaz-Arrastia et al., 2003; Harden, 2004). Further, *APOE4* is associated with an earlier age of onset for intractable seizures (Briellmann et al., 2000; Gambardella et al., 2005; Kauffman et al., 2010), greater memory dysfunction with chronic temporal lobe epilepsy (Gouras et al., 1997; Gambardella et al., 2005; Busch et al., 2007) and higher epileptiform activity after hyperventilation (Ponomareva et al., 2008). Accordingly, targeted replacement mice with *APOE4*, which are similar to the E4FAD− mice, also showed higher seizures and a faster progression through PTZ-induced seizures (Hunter et al., 2012). It is therefore conceivable that compared to *APOE3*, *APOE4* would increase the incidence of seizures and the onset of epileptiform activity in AD patients, however, there are currently no reports of such an association. Thus, further pre-clinical and clinical studies are warranted to clarify whether the neuronal changes that increase seizure phenotypes with *APOE4* is independent of AD or interact with AD pathology to manifest as a different behavior in patients.

### Seizure Incidence Is Modified by FAD Mutations but Not Sex

Previous studies in *APOE* knock-in mice have demonstrated that the effects of *APOE4* on a number of functions are particularly prominent in female mice. For example, detrimental effects of *APOE4* on learning and memory behaviors, Aβ levels, cerebrovascular function and neuroinflammation are typically higher in female mice (Balu et al., 2019). Interestingly, we did not observe an effect of sex for tonic-clonic seizures. Therefore, the changes in neuronal circuits that result in the manifestation of tonic-clonic seizures may be more proximal to the biological effects of apoE4, rather than an interaction with sex hormones.

FAD mice are known to exhibit epileptiform activity and lower threshold to seizure induction with auditory or PTZ stimuli; however, reports of tonic-clonic seizures are lacking (Palop and Mucke, 2009). 5xFAD mice were used to generate EFAD mice and are therefore similar to EFAD + mice but express mouse *APOE* and have an earlier onset of Aβ deposition. As for other FAD mice, to our knowledge there are no reports of tonic-clonic seizures in 5xFAD mice. However, when defined by electroencephalography recordings there are seizures in 5xFAD mice, possibly as early as 4 months (Abe et al., 2020), but that become prevalent at older ages (>10 months) (Paesler et al., 2015; Abe et al., 2020; Angel et al., 2020). In addition, one proposal is that the abnormal epileptiform activity predisposes 5xFAD mice to convulsive seizures with further stress, as has been demonstrated with genetic approaches (Paesler et al., 2015; Angel et al., 2020). Our data that 0% of E3FAD+ mice experience seizures are consistent with these findings, however, E4FAD+ mice did undergo a tonic-clonic seizures. These results indicate that in the presence of FAD mutations, compared to *APOE3 APOE4* may have lowered the threshold for the onset of tonic-clonic seizures, as proposed for additional stressors in 5xFAD mice.

Although our data support that compared to *APOE3*, *APOE4* is associated with higher tonic-clonic seizures, the precise interaction between *APOE4* and FAD mutations remained poorly understood. During cage change day, there were higher tonic-clonic seizures in E4FAD− mice compared to E4FAD+ mice. This result is somewhat surprising, since typically the assumption is that the combination of *APOE4* and FAD mutations would result in higher dysfunction than *APOE4* alone. One potential explanation for lower seizures in E4FAD+ mice compared to E4FAD− mice is that the FAD mutations have changed the types of seizures that are occurring with *APOE4* (e.g., to higher epileptiform activity and partial seizures as in FAD mice). On the other hand, it is possible that we have missed the detection of seizures in FAD + mice during, before or after cage changes. Alternatively, disruption of neuronal circuits involved in tonic-clonic seizures (e.g., brain stem, amygdala) with *FAD* and *APOE4* could also blunt or alter the response to stimuli produced during routine cage change. Indeed, our data obtained following PTZ injection suggest that there is higher neuronal dysfunction with FAD mutations as revealed by E4FAD+ mice exhibiting higher distribution of tonic-clonic seizures than E4FAD− mice, an effect that was also trending in *APOE3* mice.

It is also conceivable that distinct neural circuits are recruited between seizures induced during cage changes and elicited by PTZ. Stimuli produced during cage changes (sounds, new environment, handling) results in stress and anxiety in rodents, as evident from changes in behavior, hormone levels and heart rates (Duke et al., 2001; Meller et al., 2011; Rasmussen et al., 2011). In fact, placing a mouse in a new environment in ways that are similar, if not identical to the cage change procedure in our study is used as an assay of tonic-clonic seizures susceptibility (Todorova et al., 1999; Leussis and Heinrichs, 2006; Hunter et al., 2012; Qi et al., 2018). In this regard, tonic-clonic seizures during cage changes are the result of stress/anxiety signals inducing neuronal hyperexcitability. On the other hand, PTZ induces seizures by directly impacting neuronal activity, and although the precise mechanism of action is unknown (Hansen et al., 2004), it is thought to involve antagonism of GABA-A receptors. Therefore, PTZ-induced tonic-clonic seizures are a more direct maker of alterations in neuronal functional connectivity (i.e., balance of GABAergic and glutamatergic inputs). Due to the different ways that they induce seizures, there are several potential explanations for the seemingly opposite effect of FAD mutations on *APOE4* associated cage change and PTZ-induced tonic-clonic seizures. For example, the combined effects of *APOE4* and FAD may have disrupted neuronal circuits to an extent that stress cannot induce seizures, yet the remaining neurons are more sensitive to chemical-induced seizures. Alternatively, there are greater memory impairments in E4FAD+ mice than E4FAD− mice, and so EFAD− mice may anticipate what the stimuli of cage change represents. Finally, E4FAD− and E4FAD+ mice may exhibit different levels of susceptibility to stress-induced effects through aging as a result of distinct mechanisms of adaptation occurring at the neural circuit level.

### Potential Mechanisms Underlying *APOE4*-Associated Seizures

Our data also point to potential cellular mechanisms underlying the impact of *APOE4* on tonic-clonic seizure incidence *in vivo*. There are general and specific considerations for discussing this concept, all of which continue to be the focus of several research groups (reviewed in Mahley et al., 2007; Liu et al., 2013; Flowers and Rebeck, 2020). The question of how a single amino acid difference between apolipoprotein E3 (apoE3, cysteine at 112) and apolipoprotein E4 (apoE4, arginine at 112) results in modulation of such a wide range of functions in the brain is proving extremely complicated to answer and is likely context dependent. Apolipoprotein E is produced by cells in the periphery and in the brain. Within the central nervous system apoE is produced primarily by glia (astrocytes and microglia), but also by pericytes and neurons and all apoE is found on lipoprotein particles in the interstitial fluid. Therefore, one initial question surrounds the levels and lipidation state of apoE-containing lipoproteins. One suggestion is that apoE4-containing lipoproteins are lower in levels, less lipidated and/or smaller than apoE3-containing lipoproteins, which could have a profound impact on neural circuit connectivity and function. For example, changes in apoE levels and lipidation could disrupt lipoprotein functions in the interstitial fluid such as homeostasis of cholesterol and lipids, binding to debris and other substrates, and as an adaptor molecule. In addition, the structural properties of apoE4 are thought to result in altered activation and recycling of the apoE receptors in all cell types, and/or the generation of intracellular toxic apoE4 fragments in neurons. Thus, through these fundamental processes, apoE can alter neuronal network excitability directly, or indirectly through effects on inflammation, cerebrovascular function, and general homeostatic functions. Intertwined are an equally complex set of research questions that include but not limited to whether *APOE4* is a toxic gain or loss of function and whether *APOE4* imparts advantages on brain function during specific developmental windows that are detrimental in the context of aging and in response to stressors (Mahley et al., 2007; Liu et al., 2013; Flowers and Rebeck, 2020). That apoE impacts such a myriad of cell types and functions in normal and stress conditions is at the heart of why dissecting role of apoE in brain function is extremely complex. Specifically, in the context of seizures, all these changes during aging between *APOE3* and *APOE4* will likely converge to cause hyperexcitable neuronal networks in different brain regions that are important for tonic-clonic seizure manifestation.

*APOE4* has been linked to changes in neuron structure and activity in multiple brain regions including the amydagala, cortex, and the hippocampus. Of particular interest is the emerging concept that apoE4 disrupts inhibitory network function (reviewed in Najm et al., 2019). For example, in *APOE-*targeted replacement mice, compared to *APOE3*, with *APOE4* there are lower levels of GABAergic somatostatin-positive interneurons in the hippocampus, an effect that appears driven by apoE production by neurons (Najm et al., 2019). Thus, the loss of GABAergic interneurons could contribute to network hyperexcitability and higher pyramidal cell firing (Nuriel et al., 2017). In humans, there is reduced deactivation of the default mode network with *APOE4* in task-based assays (Pihlajamaki and Sperling, 2009) in association with higher hippocampal (Dickerson et al., 2005) and entorhinal activation (Bondi et al., 2005). Collectively, these observations suggest that lower GABAergic activity with *APOE4* could reduce the threshold for seizures even though *APOE4* is known to disrupt excitatory neuronal activity as well (Mahley et al., 2007; Liu et al., 2013; Flowers and Rebeck, 2020). Overall, future studies are needed to establish the extent by which *APOE4* associated seizures are directly or indirectly dependent on neuronal excitability in different cortical and subcortical brain regions.

### Limitations and Future Directions

Although we provided data on seizure incidence, an important question that remains unclear is the underlying neuronal mechanisms that are disrupted with *APOE4* to contribute to the development of seizures. Addressing this question is ultimately critical from mechanistic standpoint as well for testing in human patients. Future detailed experiments could focus on tracking epileptiform activity and seizure incidence in EFAD mice across the lifespan. We focused on older mice as we had not observed seizures during cage change day in younger mice, however there are some complications with utilizing older FAD mice (discussed in Tai et al., 2021), particularly for E4FAD+ mice. In E4FAD+ mice, Aβ pathology (soluble and extracellular) initiates around 4 months and so there is advanced pathology (e.g., Aβ, neuroinflammation, blood-brain barrier deficits) by 8 months of age. The high pathology may have altered brain function in ways that are too advanced to detect changes that are more relevant for early stages of Aβ deposition and evaluate the impact of *APOE4* and FAD on PTZ-induced seizure induction, which require younger mice. Ultimately, however, to provide more detailed mechanistic insight, pharmacological or genetic manipulations targeting either apoE, functions modulated by apoE or defined cell types and circuits could be conducted to reveal a connection with seizure activity. A discussion on the limitations of mouse models is beyond the scope of this manuscript (reviewed in Tai et al., 2021), however, it will be important to evaluate whether a seizure phenotype also manifests in APP-knock in *APOE4* mice, to validate that findings are not due to the FAD mutations.

## Conclusion

Our data demonstrate that unlike *APOE3*, *APOE4* is associated with tonic seizures, when evaluated during cage changes. There is also a higher incidence of tonic-clonic seizures with *APOE4* compared to *APOE3* following PTZ injection. However, in contrast to cage changes, the distribution of seizures to the tonic-clonic phenotype is higher with FAD mutations. These data support that *APOE4* is associated with higher tonic-clonic seizures, and that FAD mutations impact tonic-clonic seizures in a paradigm dependent manner.

## Data Availability Statement

All data supporting the conclusions of this manuscript will be made available to any qualified researcher without undue reservation.

## Ethics Statement

The animal study was reviewed and approved by the Institutional Animal Care and Use Committee at the University of Illinois at Chicago.

## Author Contributions

LL, FM, KT, and LT conceived the study, performed the experiments, and wrote the manuscript. All authors approved the submitted version.

## Conflict of Interest

The authors declare that the research was conducted in the absence of any commercial or financial relationships that could be construed as a potential conflict of interest.

## References

[B1] Abe Y. Ikegawa N. Yoshida K. Muramatsu K. Hattori S. Kawai K. (2020). Behavioral and electrophysiological evidence for a neuroprotective role of aquaporin-4 in the 5xFAD transgenic mice model. *Acta Neuropathol. Commun*. 8:67. 10.1186/s40478-020-00936-3 32398151 PMC7218576

[B2] Amatniek J. C. Hauser W. A. DelCastillo-Castaneda C. Jacobs D. M. Marder K. Bell K. (2006). Incidence and predictors of seizures in patients with Alzheimer’s disease. *Epilepsia* 47 867–872. 10.1111/j.1528-1167.2006.00554.x 16686651

[B3] Angel A. Volkman R. Royal T. G. Offen D. (2020). Caspase-6 knockout in the 5xFAD model of Alzheimer’s disease reveals favorable outcome on memory and neurological hallmarks. *Int. J. Mol. Sci*. 21:1144. 10.3390/ijms21031144 32050445 PMC7037950

[B4] Balu D. Karstens A. J. Loukenas E. Maldonado Weng J. York J. M. Valencia-Olvera A. C. (2019). The role of APOE in transgenic mouse models of AD. *Neurosci. Lett*. 707:134285. 10.1016/j.neulet.2019.134285 31150730 PMC6717006

[B5] Bernardi S. Scaldaferri N. Vanacore N. Trebbastoni A. Francia A. D’Amico A. (2010). Seizures in Alzheimer’s disease: a retrospective study of a cohort of outpatients. *Epileptic Disord*. 12 16–21. 10.1684/epd.2010.0290 20172846

[B6] Bezzina C. Verret L. Juan C. Remaud J. Halley H. Rampon C. (2015). Early onset of hypersynchronous network activity and expression of a marker of chronic seizures in the Tg2576 mouse model of Alzheimer’s disease. *PLoS One* 10:e0119910. 10.1371/journal.pone.0119910 25768013 PMC4358928

[B7] Bondi M. W. Houston W. S. Eyler L. T. Brown G. G. (2005). fMRI evidence of compensatory mechanisms in older adults at genetic risk for Alzheimer disease. *Neurology* 64 501–508. 10.1212/01.WNL.0000150885.00929.7E 15699382 PMC1761695

[B8] Bour A. Grootendorst J. Vogel E. Kelche C. Dodart J. C. Bales K. (2008). Middle-aged human apoE4 targeted-replacement mice show retention deficits on a wide range of spatial memory tasks. *Behav. Brain Res*. 193 174–182. 10.1016/j.bbr.2008.05.008 18572260

[B9] Brault V. Martin B. Costet N. Bizot J. C. Herault Y. (2011). Characterization of PTZ-induced seizure susceptibility in a down syndrome mouse model that overexpresses CSTB. *PLoS One* 6:e27845. 10.1371/journal.pone.0027845 22140471 PMC3227573

[B10] Briellmann R. S. Torn-Broers Y. Busuttil B. E. Major B. J. Kalnins R. M. Olsen M. (2000). APOE epsilon4 genotype is associated with an earlier onset of chronic temporal lobe epilepsy. *Neurology* 55 435–437. 10.1212/wnl.55.3.435 10932283

[B11] Brunetti V. D’Atri A. Della Marca G. Vollono C. Marra C. Vita M. G. (2020). Subclinical epileptiform activity during sleep in Alzheimer’s disease and mild cognitive impairment. *Clin. Neurophysiol*. 131 1011–1018. 10.1016/j.clinph.2020.02.015 32193162

[B12] Busch R. M. Lineweaver T. T. Naugle R. I. Kim K. H. Gong Y. Tilelli C. Q. (2007). ApoE-epsilon4 is associated with reduced memory in long-standing intractable temporal lobe epilepsy. *Neurology* 68 409–414. 10.1212/01.wnl.0000253021.60887.db 17283313

[B13] Cheng C. H. Liu C. J. Ou S. M. Yeh C. M. Chen T. J. Lin Y. Y. (2015). Incidence and risk of seizures in Alzheimer’s disease: a nationwide population-based cohort study. *Epilepsy Res*. 115 63–66. 10.1016/j.eplepsyres.2015.05.009 26220378

[B14] Costa C. Romoli M. Liguori C. Farotti L. Eusebi P. Bedetti C. (2019). Alzheimer’s disease and late-onset epilepsy of unknown origin: two faces of beta amyloid pathology. *Neurobiol. Aging* 73 61–67. 10.1016/j.neurobiolaging.2018.09.006 30317034

[B15] Cretin B. Sellal F. Philippi N. Bousiges O. Di Bitonto L. Martin-Hunyadi C. (2016). Epileptic prodromal Alzheimer’s disease, a retrospective study of 13 new cases: expanding the spectrum of Alzheimer’s disease to an epileptic variant? *J. Alzheimers Dis*. 52 1125–1133. 10.3233/JAD-150096 27104892

[B16] Del Vecchio R. A. Gold L. H. Novick S. J. Wong G. Hyde L. A. (2004). Increased seizure threshold and severity in young transgenic CRND8 mice. *Neurosci. Lett*. 367 164–167. 10.1016/j.neulet.2004.05.107 15331144

[B17] Diaz-Arrastia R. Gong Y. Fair S. Scott K. D. Garcia M. C. Carlile M. C. (2003). Increased risk of late posttraumatic seizures associated with inheritance of APOE epsilon4 allele. *Arch. Neurol*. 60 818–822. 10.1001/archneur.60.6.818 12810485

[B18] Dickerson B. C. Salat D. H. Greve D. N. Chua E. F. Rand-Giovannetti E. Rentz D. M. (2005). Increased hippocampal activation in mild cognitive impairment compared to normal aging and AD. *Neurology* 65 404–411. 10.1212/01.wnl.0000171450.97464.49 16087905 PMC4335677

[B19] DiFrancesco J. C. Tremolizzo L. Polonia V. Giussani G. Bianchi E. Franchi C. (2017). Adult-onset epilepsy in presymptomatic Alzheimer’s disease: a retrospective study. *J. Alzheimers Dis*. 60 1267–1274. 10.3233/JAD-170392 28968234

[B20] Duke J. L. Zammit T. G. Lawson D. M. (2001). The effects of routine cage-changing on cardiovascular and behavioral parameters in male sprague-dawley rats. *Contemp. Top. Lab. Anim. Sci*. 40 17–20. 11300670

[B21] Flowers S. A. Rebeck G. W. (2020). APOE in the normal brain. *Neurobiol. Dis*. 136:104724. 10.1016/j.nbd.2019.104724 31911114 PMC7002287

[B22] Friedman D. Honig L. S. Scarmeas N. (2012). Seizures and epilepsy in Alzheimer’s disease. *CNS Neurosci. Ther*. 18 285–294. 10.1111/j.1755-5949.2011.00251.x 22070283 PMC3630499

[B23] Gambardella A. Aguglia U. Chifari R. Labate A. Manna I. Serra P. (2005). ApoE epsilon4 allele and disease duration affect verbal learning in mild temporal lobe epilepsy. *Epilepsia* 46 110–117. 10.1111/j.0013-9580.2005.15804.x 15660776

[B24] Garcia-Cabrero A. M. Guerrero-Lopez R. Giraldez B. G. Llorens-Martin M. Avila J. Serratosa J. M. (2013). Hyperexcitability and epileptic seizures in a model of frontotemporal dementia. *Neurobiol. Dis*. 58 200–208. 10.1016/j.nbd.2013.06.005 23774255

[B25] Gouras G. K. Relkin N. R. Sweeney D. Munoz D. G. Mackenzie I. R. Gandy S. (1997). Increased apolipoprotein E epsilon 4 in epilepsy with senile plaques. *Ann. Neurol*. 41 402–404. 10.1002/ana.410410317 9066363

[B26] Grootendorst J. Bour A. Vogel E. Kelche C. Sullivan P. M. Dodart J. C. (2005). Human apoE targeted replacement mouse lines: h-apoE4 and h-apoE3 mice differ on spatial memory performance and avoidance behavior. *Behav. Brain Res*. 159 1–14. 10.1016/j.bbr.2004.09.019 15794991

[B27] Gureviciene I. Ishchenko I. Ziyatdinova S. Jin N. Lipponen A. Gurevicius K. (2019). Characterization of epileptic spiking associated with brain amyloidosis in APP/PS1 mice. *Front. Neurol*. 10:1151. 10.3389/fneur.2019.01151 31781019 PMC6861424

[B28] Hansen S. L. Sperling B. B. Sanchez C. (2004). Anticonvulsant and antiepileptogenic effects of GABAA receptor ligands in pentylenetetrazole-kindled mice. *Prog. Neuropsychopharmacol. Biol. Psychiatry* 28 105–113. 10.1016/j.pnpbp.2003.09.026 14687864

[B29] Harden C. L. (2004). The apolipoprotein E epsilon (epsilon) 4 allele is important for trauma-related epilepsy. *Epilepsy Curr*. 4 29–30. 10.1111/j.1535-7597.2004.04112.x 15346143 PMC324582

[B30] Hauser W. A. Morris M. L. Heston L. L. Anderson V. E. (1986). Seizures and myoclonus in patients with Alzheimer’s disease. *Neurology* 36 1226–1230. 10.1212/wnl.36.9.1226 3092131

[B31] Hesdorffer D. C. Hauser W. A. Annegers J. F. Kokmen E. Rocca W. A. (1996). Dementia and adult-onset unprovoked seizures. *Neurology* 46 727–730. 10.1212/wnl.46.3.727 8618673

[B32] Hirsch L. J. (2007). ApoE, MemorE, and EpilepsE. *Epilepsy Curr*. 7 149–150. 10.1111/j.1535-7511.2007.00208.x 18049720 PMC2096726

[B33] Hunter J. M. Cirrito J. R. Restivo J. L. Kinley R. D. Sullivan P. M. Holtzman D. M. (2012). Emergence of a seizure phenotype in aged apolipoprotein epsilon 4 targeted replacement mice. *Brain Res*. 1467 120–132. 10.1016/j.brainres.2012.05.048 22682924

[B34] Imfeld P. Bodmer M. Schuerch M. Jick S. S. Meier C. R. (2013). Seizures in patients with Alzheimer’s disease or vascular dementia: a population-based nested case-control analysis. *Epilepsia* 54 700–707. 10.1111/epi.12045 23215680

[B35] Irizarry M. C. Jin S. He F. Emond J. A. Raman R. Thomas R. G. (2012). Incidence of new-onset seizures in mild to moderate Alzheimer disease. *Arch. Neurol*. 69 368–372. 10.1001/archneurol.2011.830 22410444 PMC3622046

[B36] Kauffman M. A. Consalvo D. Moron D. G. Lereis V. P. Kochen S. (2010). ApoE epsilon4 genotype and the age at onset of temporal lobe epilepsy: a case-control study and meta-analysis. *Epilepsy Res*. 90 234–239. 10.1016/j.eplepsyres.2010.05.007 20554432

[B37] Kazim S. F. Chuang S. C. Zhao W. Wong R. K. Bianchi R. Iqbal K. (2017). Early-onset network hyperexcitability in presymptomatic Alzheimer’s disease transgenic mice is suppressed by passive immunization with anti-human APP/Abeta antibody and by mGluR5 blockade. *Front. Aging Neurosci*. 9:71. 10.3389/fnagi.2017.00071 28392767 PMC5364175

[B38] Keret O. Hoang T. D. Xia F. Rosen H. J. Yaffe K. (2020). Association of late-onset unprovoked seizures of unknown etiology with the risk of developing dementia in older veterans. *JAMA Neurol*. 77 710–715. 10.1001/jamaneurol.2020.0187 32150220 PMC7063560

[B39] Lam A. D. Noebels J. (2020). Night watch on the titanic: detecting early signs of Epileptogenesis in Alzheimer disease. *Epilepsy Curr*. 20 369–374. 10.1177/1535759720964775 33081517 PMC7818196

[B40] Lam A. D. Sarkis R. A. Pellerin K. R. Jing J. Dworetzky B. A. Hoch D. B. (2020). Association of epileptiform abnormalities and seizures in Alzheimer disease. *Neurology* 95 e2259–e2270. 10.1212/WNL.0000000000010612 32764101 PMC7713786

[B41] Leussis M. P. Heinrichs S. C. (2006). Routine tail suspension husbandry facilitates onset of seizure susceptibility in EL mice. *Epilepsia* 47 801–804. 10.1111/j.1528-1167.2006.00525.x 16650149

[B42] Liang Y. Zhou Z. Wang H. Cheng X. Zhong S. Zhao C. (2019). Association of apolipoprotein E genotypes with epilepsy risk: a systematic review and meta-analysis. *Epilepsy Behav*. 98(Pt A), 27–35. 10.1016/j.yebeh.2019.06.015 31299529

[B43] Liu C. C. Liu C. C. Kanekiyo T. Xu H. Bu G. (2013). Apolipoprotein E and Alzheimer disease: risk, mechanisms and therapy. *Nat. Rev. Neurol*. 9 106–118. 10.1038/nrneurol.2012.263 23296339 PMC3726719

[B44] Lozsadi D. A. Larner A. J. (2006). Prevalence and causes of seizures at the time of diagnosis of probable Alzheimer’s disease. *Dement. Geriatr. Cogn. Disord*. 22 121–124. 10.1159/000093664 16733353

[B45] Mahley R. W. Huang Y. Weisgraber K. H. (2007). Detrimental effects of apolipoprotein E4: potential therapeutic targets in Alzheimer’s disease. *Curr. Alzheimer Res*. 4 537–540. 10.2174/156720507783018334 18220516

[B46] McAreavey M. J. Ballinger B. R. Fenton G. W. (1992). Epileptic seizures in elderly patients with dementia. *Epilepsia* 33 657–660. 10.1111/j.1528-1157.1992.tb02343.x 1628580

[B47] Meller A. Kasanen I. Ruksenas O. Apanaviciene N. Baturaite Z. Voipio H. M. (2011). Refining cage change routines: comparison of cardiovascular responses to three different ways of cage change in rats. *Lab. Anim*. 45 167–173. 10.1258/la.2011.010134 21498640

[B48] Mendez M. F. Catanzaro P. Doss R. C. Raul A. Frey W. H. II (1994). Seizures in Alzheimer’s disease: clinicopathologic study. *J. Geriatr. Psychiatry Neurol*. 7 230–233. 10.1177/089198879400700407 7826492

[B49] Minkeviciene R. Rheims S. Dobszay M. B. Zilberter M. Hartikainen J. Fulop L. (2009). Amyloid beta-induced neuronal hyperexcitability triggers progressive epilepsy. *J. Neurosci*. 29 3453–3462. 10.1523/JNEUROSCI.5215-08.2009 19295151 PMC6665248

[B50] Najm R. Jones E. A. Huang Y. (2019). Apolipoprotein E4, inhibitory network dysfunction, and Alzheimer’s disease. *Mol. Neurodegener*. 14:24. 10.1186/s13024-019-0324-6 31186040 PMC6558779

[B51] Nuriel T. Angulo S. L. Khan U. Ashok A. Chen Q. Figueroa H. Y. (2017). Neuronal hyperactivity due to loss of inhibitory tone in APOE4 mice lacking Alzheimer’s disease-like pathology. *Nat. Commun*. 8:1464. 10.1038/s41467-017-01444-0 29133888 PMC5684208

[B52] Paesler K. Xie K. Hettich M. M. Siwek M. E. Ryan D. P. Schroder S. (2015). Limited effects of an eIF2alphaS51A allele on neurological impairments in the 5xFAD mouse model of Alzheimer’s disease. *Neural Plast*. 2015:825157. 10.1155/2015/825157 25883808 PMC4391319

[B53] Palop J. J. Chin J. Roberson E. D. Wang J. Thwin M. T. Bien-Ly N. (2007). Aberrant excitatory neuronal activity and compensatory remodeling of inhibitory hippocampal circuits in mouse models of Alzheimer’s disease. *Neuron* 55 697–711. 10.1016/j.neuron.2007.07.025 17785178 PMC8055171

[B54] Palop J. J. Mucke L. (2009). Epilepsy and cognitive impairments in Alzheimer disease. *Arch. Neurol*. 66 435–440. 10.1001/archneurol.2009.15 19204149 PMC2812914

[B55] Pandis D. Scarmeas N. (2012). Seizures in Alzheimer disease: clinical and epidemiological data. *Epilepsy Curr*. 12 184–187. 10.5698/1535-7511-12.5.184 23118603 PMC3482722

[B56] Pihlajamaki M. Sperling R. A. (2009). Functional MRI assessment of task-induced deactivation of the default mode network in Alzheimer’s disease and at-risk older individuals. *Behav. Neurol*. 21 77–91. 10.3233/BEN-2009-0231 19847047 PMC5450588

[B57] Ponomareva N. V. Korovaitseva G. I. Rogaev E. I. (2008). EEG alterations in non-demented individuals related to apolipoprotein E genotype and to risk of Alzheimer disease. *Neurobiol. Aging* 29 819–827. 10.1016/j.neurobiolaging.2006.12.019 17293007

[B58] Qi J. Kim M. Sanchez R. Ziaee S. M. Kohtz J. D. Koh S. (2018). Enhanced susceptibility to stress and seizures in GAD65 deficient mice. *PLoS One* 13:e0191794. 10.1371/journal.pone.0191794 29377906 PMC5788371

[B59] Rao S. C. Dove G. Cascino G. D. Petersen R. C. (2009). Recurrent seizures in patients with dementia: frequency, seizure types, and treatment outcome. *Epilepsy Behav*. 14 118–120. 10.1016/j.yebeh.2008.08.012 18782632 PMC2875670

[B60] Rasmussen S. Miller M. M. Filipski S. B. Tolwani R. J. (2011). Cage change influences serum corticosterone and anxiety-like behaviors in the mouse. *J. Am. Assoc. Lab. Anim. Sci*. 50 479–483. 21838975 PMC3148651

[B61] Risse S. C. Lampe T. H. Bird T. D. Nochlin D. Sumi S. M. Keenan T. (1990). Myoclonus, seizures, and paratonia in Alzheimer disease. *Alzheimer Dis. Assoc. Disord*. 4 217–225. 10.1097/00002093-199040400-00003 2264979

[B62] Rodriguez G. A. Burns M. P. Weeber E. J. Rebeck G. W. (2013). Young APOE4 targeted replacement mice exhibit poor spatial learning and memory, with reduced dendritic spine density in the medial entorhinal cortex. *Learn. Mem*. 20 256–266. 10.1101/lm.030031.112 23592036 PMC3630489

[B63] Romanelli M. F. Morris J. C. Ashkin K. Coben L. A. (1990). Advanced Alzheimer’s disease is a risk factor for late-onset seizures. *Arch. Neurol*. 47 847–850. 10.1001/archneur.1990.00530080029006 2375689

[B64] Sarkis R. A. Dickerson B. C. Cole A. J. Chemali Z. N. (2016). Clinical and neurophysiologic characteristics of unprovoked seizures in patients diagnosed with dementia. *J. Neuropsychiatry Clin. Neurosci*. 28 56–61. 10.1176/appi.neuropsych.15060143 26404175

[B65] Scarmeas N. Honig L. S. Choi H. Cantero J. Brandt J. Blacker D. (2009). Seizures in Alzheimer disease: who, when, and how common? *Arch. Neurol*. 66 992–997. 10.1001/archneurol.2009.130 19667221 PMC2768279

[B66] Siwek M. E. Muller R. Henseler C. Trog A. Lundt A. Wormuth C. (2015). Altered theta oscillations and aberrant cortical excitatory activity in the 5XFAD model of Alzheimer’s disease. *Neural Plast*. 2015:781731. 10.1155/2015/781731 25922768 PMC4398951

[B67] Tai L. M. Balu D. Avila-Munoz E. Abdullah L. Thomas R. Collins N. (2017). EFAD transgenic mice as a human APOE relevant preclinical model of Alzheimer’s disease. *J. Lipid Res*. 58 1733–1755. 10.1194/jlr.R076315 28389477 PMC5580905

[B68] Tai L. M. Maldonado Weng J. LaDu M. J. Brady S. T. (2021). Relevance of transgenic mouse models for Alzheimer’s disease. *Prog. Mol. Biol. Transl. Sci*. 177 1–48. 10.1016/bs.pmbts.2020.07.007 33453936 PMC8163103

[B69] Tai L. M. Thomas R. Marottoli F. M. Koster K. P. Kanekiyo T. Morris A. W. (2016). The role of APOE in cerebrovascular dysfunction. *Acta Neuropathol*. 131 709–723. 10.1007/s00401-016-1547-z 26884068 PMC4837016

[B70] Thomas R. Morris A. W. J. Tai L. M. (2017). Epidermal growth factor prevents APOE4-induced cognitive and cerebrovascular deficits in female mice. *Heliyon* 3:e00319. 10.1016/j.heliyon.2017.e00319 28626809 PMC5463012

[B71] Todorova M. T. Burwell T. J. Seyfried T. N. (1999). Environmental risk factors for multifactorial epilepsy in EL mice. *Epilepsia* 40 1697–1707. 10.1111/j.1528-1157.1999.tb01586.x 10612332

[B72] Van Erum J. Valkenburg F. Van Dam D. De Deyn P. P. (2020). Pentylenetetrazole-induced seizure susceptibility in the Tau58/4 transgenic mouse model of tauopathy. *Neuroscience* 425 112–122. 10.1016/j.neuroscience.2019.11.007 31785360

[B73] Villasana L. Acevedo S. Poage C. Raber J. (2006). Sex- and APOE isoform-dependent effects of radiation on cognitive function. *Radiat. Res*. 166 883–891. 10.1667/RR0642.1 17149978

[B74] Volicer L. Smith S. Volicer B. J. (1995). Effect of seizures on progression of dementia of the Alzheimer type. *Dementia* 6 258–263. 10.1159/000106956 8528372

[B75] Vossel K. A. Beagle A. J. Rabinovici G. D. Shu H. Lee S. E. Naasan G. (2013). Seizures and epileptiform activity in the early stages of Alzheimer disease. *JAMA Neurol*. 70 1158–1166. 10.1001/jamaneurol.2013.136 23835471 PMC4013391

[B76] Vossel K. A. Ranasinghe K. G. Beagle A. J. Mizuiri D. Honma S. M. Dowling A. F. (2016). Incidence and impact of subclinical epileptiform activity in Alzheimer’s disease. *Ann. Neurol*. 80 858–870. 10.1002/ana.24794 27696483 PMC5177487

[B77] Westmark C. J. Westmark P. R. Malter J. S. (2010). Alzheimer’s disease and down syndrome rodent models exhibit audiogenic seizures. *J. Alzheimers Dis*. 20 1009–1013. 10.3233/JAD-2010-100087 20413855 PMC2915889

[B78] Yagi H. Takamura Y. Yoneda T. Konno D. Akagi Y. Yoshida K. (2005). Vlgr1 knockout mice show audiogenic seizure susceptibility. *J. Neurochem*. 92 191–202. 10.1111/j.1471-4159.2004.02875.x 15606908

[B79] Youmans K. L. Tai L. M. Nwabuisi-Heath E. Jungbauer L. Kanekiyo T. Gan M. (2012). APOE4-specific changes in Abeta accumulation in a new transgenic mouse model of Alzheimer disease. *J. Biol. Chem*. 287 41774–41786. 10.1074/jbc.M112.407957 23060451 PMC3516726

[B80] Zaldua S. Damen F. C. Pisharody R. Thomas R. Fan K. D. Ekkurthi G. K. (2020). Epidermal growth factor treatment of female mice that express APOE4 at an age of advanced pathology mitigates behavioral and cerebrovascular dysfunction. *Heliyon* 6:e03919. 10.1016/j.heliyon.2020.e03919 32478184 PMC7251379

